# Psychophysical assessment of face perception deficits in adults with amblyopia through top-down and bottom-up visual processing pathways

**DOI:** 10.3389/fnins.2025.1548243

**Published:** 2025-05-21

**Authors:** Xiaolu Ming, Gantian Huang, Meng Liao, Ping Jiang, Longqian Liu

**Affiliations:** ^1^Department of Ophthalmology, West China Hospital, Sichuan University, Chengdu, China; ^2^Laboratory of Optometry and Vision Sciences, West China Hospital, Sichuan University, Chengdu, China; ^3^Functional and Molecular Imaging Key Laboratory of Sichuan Province, Department of Radiology and Huaxi MR Research Center (HMRRC), West China Hospital, Sichuan University, Chengdu, China; ^4^West China Medical Publishers, West China Hospital, Sichuan University, Chengdu, China; ^5^Research Unit of Psychoradiology, Chinese Academy of Medical Sciences, Chengdu, China

**Keywords:** amblyopia, face perception, psychophysics, visual cognition, top-down

## Abstract

**Purpose:**

This study aims to investigate face perception ability in adult patients with amblyopia.

**Methods:**

We conducted two psychophysical experiments. The Face-detection task involved 25 amblyopic patients and 25 healthy controls, using face stimulation at 6 stimulation intensities. The Toast task included 16 amblyopic patients and 15 healthy controls, with pure noise images and semantic cues designed to induce face perception. We recorded accuracy and reaction times (RT) and used the Kruskal-Wallis test with Wilcoxon comparisons to analyze group differences.

**Results:**

In the Face-detection task, amblyopic eyes (AE) exhibited significantly higher face detection thresholds than healthy controls (*P* < 0.05), indicating face detection deficit. AE showed lower accuracy at 20 and 67% stimulation intensities compared with HC and fellow eyes (*Ps* < 0.01). The Toast task revealed no significant differences in false alarm rate or RT were observed between groups (*P* > 0.1).

**Conclusion:**

This study shows that patients with amblyopia have impaired face perception, with higher threshold and lower accuracy, especially under lower stimulation conditions. These findings highlight the need for further research to understand the neural basis of these deficits and explore potential treatments. Ultimately, these study results may provide valuable insights and fill an important gap in the psychophysical understanding of amblyopia.

## 1 Introduction

Amblyopia is a neurodevelopmental disorder characterized by monocular or binocular visual acuity reduction due to prolonged visual deprivation or imbalanced visual input stimulation during early childhood, and it is one of the most common cause of monocular visual impairment, affecting 3–5% of the world population (Birch et al., 2022; Cakir et al., 2024; Holmes and Clarke, 2006). If not detected and treated early, amblyopic children may suffer irreversible vision impairment, resulting in permanent visual impairment (Birch et al., 2013), resulting in many individuals continue to experience persistent visual deficits into adulthood (Li R. et al., 2011). These deficits extend to many aspects of binocular functions, such as contrast sensitivity, stereopsis and motion perception (Birch et al., 2013; Dulaney et al., 2023; Giaschi et al., 2024; Jeon and Choi, 2017; Levi, 2013). Recent evidence also suggests that amblyopia involves functional abnormalities in early visual pathways. Neuroimaging studies have reported reduced responses in the parvocellular layers of the lateral geniculate nucleus (LGN) and decreased feedforward connectivity from LGN to V1 when the amblyopic eye is stimulated (Li X. et al., 2011; Wen et al., 2021). These findings indicate that deficits may originate as early as the retina–LGN–V1 circuit and may limit the quality of visual information reaching cortical areas involved in perception. Recent research in amblyopia have revealed that this visual impairment is not limited to basic visual functions but also extends to higher-order visual processing tasks, such as visual attention, memory and social cognition (Black et al., 2021; Mao et al., 2024; Orquin et al., 2021; Verghese et al., 2019; Wang et al., 2017). These findings suggest that impacts of amblyopia are more pervasive than previously understood, which could lead to significant psychosocial impacts, including reduced social interactions, anxiety over potential vision loss in the fellow eye, limited career opportunities, etc. (Liinamaa et al., 2023; Yong et al., 2022). Existing research further suggests that visual functioning should be conceptualized across multiple levels, encompassing not only structural and functional integrity of the eye, but also task-specific abilities and societal outcomes. This multidimensional perspective underscores the need to assess amblyopia-related impairments beyond conventional visual metrics (Colenbrander and Fletcher, 2018; Colenbrander, 2024).

Face perception is a core component of social cognition, essential for interpreting identity, emotion, and intention in daily life. It is supported by well-established neural circuits and widely validated experimental paradigms, making it an ideal model for investigating high-level visual processing. However, there is currently a lack of direct evidence indicating that individuals with amblyopia exhibit systematic impairments in social cognitive functions. Whether they experience perceptual deficits in complex socially visual stimuli, such as faces, remains open and underexplored. Face perception is a crucial cognitive function in humans and is primarily supported by two processing pathways: the bottom-up and top-down pathways (Fan et al., 2020; Haxby et al., 2000). Bottom-up pathway plays an important role in processing basic facial features such as identity, gender, and race. In addition to identity recognition, bottom-up pathway is also involved in processing emotional expressions (Kay and Yeatman, 2017; Woolnough et al., 2022; Yovel and Kanwisher, 2008). Core face areas includes the fusiform face area (FFA) in the ventral occipitotemporal region (Kanwisher et al., 1997), the occipital face area (OFA) in the ventral occipital cortex (Gauthier et al., 2000), and the superior temporal sulcus (STS) (Materna et al., 2008). The bottom-up pathway begins in the primary visual cortex (V1) and to the inferior temporal cortex. As the beginning of the bottom-up pathway, the primary visual cortex (V1) plays a critical role in encoding early visual information. Neuroimaging studies have revealed structural and functional abnormalities in V1 among individuals with amblyopia, which are likely to disrupt the transmission of visual signals to higher-order regions involved in face perception. However, current research on amblyopia has predominantly focused on deficits in basic visual functions such as visual acuity, contrast sensitivity, and stereopsis. Although these studies reflect deficits in early visual processing, they are based on simple stimuli and may not generalize to the perception of complex, socially relevant inputs. In particular, the impact of amblyopia on face perception remains poorly understood.

Face perception is not only driven by visual input face stimulation from the bottom-up, but it is also strongly influenced by the top-down signals (Hadders-Algra, 2022). The orbitofrontal cortex (OFC) can be activated early and guide face processing by sending signals to the FFA. This top-down signal is especially important in tasks that involve social attribute judgment (Cushing et al., 2018). Top-down processing also helps to bring up mental images, memories, prior knowledge, expectations, and attention to support recognition, especially when the visual input is incomplete or unclear (Bi et al., 2024; Buschman and Miller, 2007; Pardi et al., 2020). Specifically, it remains unclear whether they can rely solely on prior knowledge and subjective inference to maintain perceptual performance comparable to that of individuals with normal vision, particularly under blurring conditions of image. To fill this gap, we designed the Toast task and investigated the ability of amblyopic individuals to perceive faces under conditions of pure noise.

Studies have used blurred faces or face-like paradigms to find top-down effects in face perception. However, these paradigms often still contain facial structure, so it is difficult to rule out the role of bottom-up input or attention (Akdeniz et al., 2018; Andrews et al., 2002; Heekeren et al., 2008; Owen et al., 2012; Rorie and Newsome, 2005; Tong et al., 1998; Wardle et al., 2020). In our study, we designed the toast task to eliminate the influence of the bottom-up pathway activation. The task included a training phase with face pictures, followed by a test phase where all images were made of pure noise and contained no real face features, and patients with amblyopia were asked to decide whether a face was present. This task helped us find face perception through top-down pathway alone and it can allow us to explore whether adult patients with amblyopia rely on top-down pathway to support face perception. Research on face perception ability in patients with amblyopia has not received widespread attention, and there is a lack of literature supporting that these patients experience deficits in face perception. While studies have explored face perception ability in patients with deprivation amblyopia caused by congenital cataracts, these investigations have typically not differentiated the roles of bottom-up and top-down processing pathways in face perception (de Heering and Maurer, 2014; Kelly et al., 2012).

Above all, the lack of early visual experience in individuals with amblyopia not only impairs low-level visual functions but also lead to deficits in higher-order visual processing tasks, such as face perception (Bankó et al., 2013a; Jia et al., 2022; Liu et al., 2024). Existing research has mainly focused on impairments in the primary visual cortex, offering limited insight into how amblyopia affects the perception of complex, socially meaningful stimuli like faces. Most current studies use simple, high-contrast shapes, which do not reflect the ambiguity of real-world input often influenced by blur, shadow, and background noise. To address this gap, this study aims to investigate face perception deficits in adults with amblyopia in Face detection task with 6 levels of stimulus intensities. We developed the Toast task which eliminated bottom-up signals by presenting pure noise images after training. After a training phase with increasing difficulty, participants are tested on their ability to perceive faces in pure noise images. To further explore the role of prior information in modulating face perception, the experiment includes 3 semantic instructions designed to induce expectation regarding the presence of a face.

## 2 Materials and methods

### 2.1 Participants

This study adhered to the principles of the Declaration of Helsinki and received approval from the Ethics Committee of West China Hospital, Sichuan University. Written informed consent was obtained from each subject before participation. This experiment has been registered with the China Clinical Trial Center (ChiCTR2400092424). The face-detection task included 25 patients with amblyopia and 25 healthy controls (HC). The toast task included 16 patients and 15 healthy controls (HCs), all of whom were participants in the face-detection task. Detailed information (Table 1) on the subjects and clinical details about patients with amblyopia (Table 2) in Face-detection task is presented below. The sample size calculation process is outlined in Supplementary material S1. All amblyopic participants had a confirmed diagnosis and underwent comprehensive ocular examinations conducted at the time of the experiment. Inclusion criteria for patients were: (1) amblyopia with anisometropia, strabismus, or both; (2) age between 18 and 50 years; (3) right-handedness; and (4) we included amblyopic subjects who had best-corrected visual acuity of 0.15 logMAR or worse in the amblyopic eye, normal vision in the fellow eye and an interocular difference of two or more lines, or best-corrected visual acuity worse than 0.15 logMAR in both eyes (Cruz et al., 2023; Rakshit et al., 2024). All health controls (HC) met the following criteria: (1) age between 18 and 50 years; (2) right-handedness; and (3) normal binocular visual functions and corrected logMAR visual acuity must reach 0.0. Exclusion criteria for both groups included: (1) any organic eye disease; (2) neurological disorders; (3) psychiatric, neurological disorders, or intellectual disabilities.

**TABLE 1:** Clinical characteristics of healthy control group, amblyopic eye and fellow eye in amblyopia group.

**Face-detection task**				
**Clinical details**	**Healthy controls**	**Amblyopic eye**	**Fellow eye**	***P***
Number	25	25		
Male gender, *n* (%)	7 (28%)	11 (44%)		0.189
Age (years)	27.12 ± 4.82	24.08 ± 2.53		0.017
Spherical equivalent (diopters)	−2.75 ± 0.44	2.24 ± 0.53	−1.39 ± 0.55	<0.001
BCVA (logMAR)	0.03 ± 0.014	0.73 ± 0.07	0.07 ± 0.032	<0.001
Axial length (mm)	24.9 ± 0.25	22.45 ± 0.23	23.81 ± 0.22	<0.001
**Toast task**				
**Clinical details**	**Healthy controls**	**Amblyopia eye**	**Fellow eye**	
Number	15	16		
Male sex, *n* (%)	8 (50%)	5 (31%)		0.189
Age (years)	24.13 ± 3.23	26.06 ± 4.34		0.17
Spherical equivalent (diopters)	−2.25 ± 1.53	3.3 ± 2.38	−0.39 ± 2.61	<0.001
BCVA (logMAR)	0.01 ± 0.07	0.72 ± 0.42	0.03 ± 0.094	<0.001
Axial length (mm)	24.78 ± 1.13	22.32 ± 1.12	23.59 ± 1.10	<0.001

BCVA, best-corrected visual acuity; SD, standard deviation.

**TABLE 2:** Clinical details of amblyopic subjects.

		Refraction		logMAR VA		Squint
**Subject**	**Age/gender**	**RE**	**LE**	**RE**	**LE**	
A1	36/F	−4.25/−1.0 110°	+0.75	0.54	0.70	ø
A2	29/F	+3.0/−0.5 0°	−2.75/−1.5 16°	0.56	0.44	ø
A3	22/F	−2.0/−0.75 166°	+3.75/−0.25 22°	0.14	0.54	ø
A4	28/F	−2.25/−0.5 45°	+4.75/−2.75 0°	−0.14	0.70	ø
A5	35/F	+5.0/−1.5 3°	+1.0	0.52	0.12	ø
A6	22/M	−6.0/−1.0 175°	−4.0/−1.5 165°	−0.02	0.60	ø
A7	32/F	+1.75/−3.0 180°	−0.75	1.20	0.02	ø
A8	32/F	−2.25/−0.25 90°	+2.0/−1.25 60°	−0.10	0.66	ø
A9	30/M	+7.0/−1.0 135°	−2.75/−0.5 175°	0.96	−0.04	ø
A10	25/M	+4.0/−0.75 100°	−0.5 25°	0.48	0.08	ø
A11	21/F	−0.75	+5.0/−0.25 55°	−0.04	0.60	ø
A12	21/F	+5.0/−5.0 160°	−0.5	0.54	0.04	ø
A13	28/M	+0.75/−1.5 180°	+0.25/−3.5 175°	0.46	0.08	ø
S1	18/F	+2.25/−0.25 90°	+2.0/−0.25 10°	0.52	0.12	D4Δ, N11Δ ET (R/L 1)
S2	25/M	−0.5/−0.5 150°	−0.5	0.40	0.04	D23Δ, N19Δ ET (L/R 3)
S3	31/F	+5.50/−0.5 50°	+6.5	0.10	0.94	D14Δ, N28Δ ET
S4	32/F	Plano	−0.5 90°	0.00	0.80	N20Δ XT (R/L 20)
SA1	26/M	−1.0 166°	−3.0	0.44	0.04	D7Δ, N13Δ ET (L/R 4)
SA2	21/F	+1.25 /−0.5 155°	−2.5	0.70	0.12	D4Δ, N15Δ ET
SA3	33/M	Plano	−4.0/−1.50 165°	1.00	0.20	D15Δ, N15Δ ET
SA4	29/F	Plano	+5.0/−1.25 25°	0.00	0.72	D10Δ, N4Δ XT
SA5	28/F	−2.0/−0.5 135°	+1.0/−0.75 10°	0.10	1.60	D42Δ, N15Δ ET
SA6	24/F	+4.75/−1.25 100°	+6.0/−0.5 30°	−0.04	0.42	D15Δ, N15Δ ET
SA7	26/F	+3.75	−1.0	1.60	−0.16	D20Δ, N30Δ ET
SA8	24/F	+1.25	−3.0/−0.5 145°	0.64	0.02	D9Δ, N23Δ ET

A, anisometropic; S, strabismic; SA, strabismic and anisometropic; RE, right eye; LE, left eye; VA, visual acuity; D, distant; N, near; ET, esotropia; XT, exotropia. One subject (SA4) was reclassified as having deprivation amblyopia due to the presence of a persistent pupillary membrane in the amblyopic eye.

Patients with amblyopia were consecutively recruited for the face-detection task between July 2023 and June 2024 from the Department of Ophthalmology, West China Hospital, Sichuan University. The examinations included decimal visual acuity, fundus examination, objective refraction, prism test, and visual function assessment. Among patients with amblyopia in Face-detection task, 13 had anisometropic amblyopia, 8 had mixed amblyopia, and 4 had strabismic amblyopia (1 with exotropia and 3 with esotropia). In Toast task, 7 had anisometropic amblyopia, 3 had strabismic amblyopia (3 with esotropia) and 6 had mixed amblyopia. The toast task was conducted using the same inclusion and exclusion criteria as the face-detection task. Participants for the toast task were recruited between November 2023 and June 2024.

### 2.2 Apparatus

**FIGURE 1:**
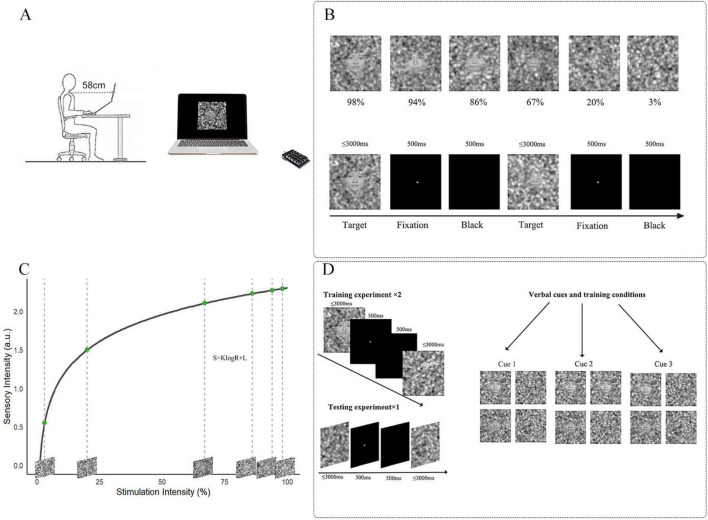
Overview of stimulus intensities and experimental process. **(A)** Participants were required to make selections using a numerical keypad. **(B)** Top line: Selection of 6 different stimulation intensity images; bottom line: How is the threshold task presented on the computer. **(C)** Stimulation Intensities Derived from Fechner’s Law. The formula of Fechner’s Law is given by *S* = *KlogR* + *L*, where *S* Represents sensory intensity, *R* is the stimulation intensity, *K* is a constant related to the sensitivity of perception, and *L* is an offset constant. Six stimulation intensities that conform to the Fechner distribution were calculated using MATLAB, corresponding with the topline **(B)**. **(D)** Left: Based on the results of the threshold task, the training groups used images with 98 and 86% stimulation intensity. The ratio of face images to pure noise images in the training groups matched the verbal induction ratio provided before the experiment. The testing group consisted entirely of pure noise images. Right: three different Instructions provided by the experimenter before the experiment.

Stimulus images were generated using MATLAB 2022b (The MathWorks Corp., Natick, MA, USA), and the experimental paradigm was implemented in E-Prime 3.0. The experiment was conducted on a Lenovo Think Book 14p Gen 2 laptop, with stimuli presented on its 14-inch display (300 mm × 188 mm), which had a spatial resolution of 2,240 × 1,400 pixels and a refresh rate of 90 Hz. Participants viewed the stimuli monocularly with optimal optical correction in a dark room, with the non-tested eye occluded by an opaque patch. E-Prime recorded reaction times (RT) and key presses throughout the experiment. Participants viewed the stimuli from 58 cm and were given a 3-s window to respond, during which they were instructed to react as quickly as possible (Figure 1A).

## 3 Experiment 1: face-detection task

### 3.1 Stimuli

In this experiment, 20 neural facial pictures (10 male and 10 female) were randomly selected from the Chinese Facial Affective Picture System (CFAPS) (Zhao et al., 2016). All pictures were grayscales with a resolution of 480 × 480 pixels. Picture generation was conducted using MATLAB R2022b, based on the fase-noise linear blending method proposed by Liu et al. (2014). Each face picture was linearly combined with pure noise images to create stimuli with 6 intensity levels, allowing for quantitative manipulation of face perception ability. The pure noise was generated using Gaussian blobs with 3 standard deviations (m_1_ = 64, m_2_ = 256, m_3_ = 1,024), which were weighted and summed as follows: m1 × 1 + m2 × 5 + m3 × 25. A blending parameter **λ** was introduced to control the ratio between the face image and the noise, where a smaller λ (closer to 0) indicates a clearer facial structure, and a larger λ (closer to 1) indicates a higher proportion of noise, making the face harder to recognize. According to Liu et al. (2014), **λ = 0.3** corresponds to an “easily perceptible face,” while **λ = 0.9997** corresponds to a “barely perceptible face.” Building upon this framework, we further applied Fechner’s Law to design 6 different stimulus intensity levels, extending beyond a simple binary classification of face perception. Fechner’s Law posits that the perceived intensity (**S**) of a stimulus is a logarithmic function of its physical intensity (**R**), suggesting that human sensation increases with the physical stimulus, but in a logarithmic rather than linear manner. The formula is as follows:


S=K⁢l⁢o⁢g⁢R+L

In this formula, *S* represents the perceived intensity, *R* denotes the physical stimulus intensity, and *K* and *L* are numbers. Based on this law and in conjunction with the parameter calculation formula proposed by Liu et al., the present study adopted logarithmic sampling for the setting of the λ values, resulting in intensity levels of 98, 94, 86, 67, 20, and 3% which were used in this study (Figures 1B,C). The Face-detection task included all stimulation intensities, with every picture containing a face.

### 3.2 Procedure

Participants viewed the stimulus image at 58 cm (Ferrari et al., 2018; Renzi et al., 2013). This task employed a block-related experimental paradigm. Before the experiment, each participant viewed a sample stimulus image to ensure clear monocular vision. Participants did not receive any information that could lead to expectations. The Face-detection task was conducted over two sessions, with each eye tested separately, and all images were presented in a random order. The Face-detection task comprised 4 blocks, each containing 120 trials (20 facial images × 6 stimulation intensities). Each trial began with a 5,000 ms instruction phase, followed by a 5,000 ms fixation cross (“+”). Subsequently, a face stimulation image was presented for up to 3,000 ms, during which participants were required to respond, and the trial advanced to the next phase immediately after a response was made. This was followed by a 500 ms fixation cross (“+”) and a 500 ms blank black screen. During this task, participants were instructed to press “1” if they detected a face in the image and “2” if they did not. Stimulus presentation was randomized.

### 3.3 Analysis

#### 3.3.1 Data analysis

We collected accuracy (ACC) and reaction time (RT) data from each participant across two sessions and calculated the mean and standard deviation (SD) for both ACC and RT in the AE, fellow eye (FE) in amblyopia group and HC groups under six different stimulus intensities, subjects with amblyopia were tested with AE and FE eyes separately. Since the data were non-normally distributed, we used the Kruskal-Wallis test for non-parametric group comparisons. Specifically, Wilcoxon signed-rank tests were used for comparisons between AE and FE, while Mann-Whitney U tests were used for comparisons between AE and HC, and between FE and HC, we then performed pairwise comparisons using the Wilcoxon test or Mann-Whitney U test and applied Bonferroni correction for multiple comparisons. Group (amblyopia vs. healthy controls) served as the between-subject factor, and stimulus intensity as the within-subject factor. Since both eyes in HC group showed similar performance, the averaged result from both eyes were used in all analyses. As shown in the results presented in Table 1, there is a significant difference in age between amblyopic patients and normal subjects in the Face-detection task (*P* = 0.017). To address the potential age difference between the amblyopic and control groups, we conducted a stratified analysis based on age. Detailed methods and results are provided in Supplementary material S2.

#### 3.3.2 Fitting function

To compare the threshold differences among AE, FE in amblyopia group and healthy control group and visually illustrate the psychophysical curve trends, we fitted 3 representative psychometric curve individually with a logistic function constrained between 0 and 1. For each group of 25 participants, we first fitted individual curves, then calculated the threshold at which each participant achieved 50% accuracy in face detection and analyzed the differences in these thresholds between groups. We averaged the thresholds across all participants within each group to obtain group mean threshold of face detections.

### 3.4 Results

#### 3.4.1 Individual threshold of face-detection tasks at six intensities

**FIGURE 2:**
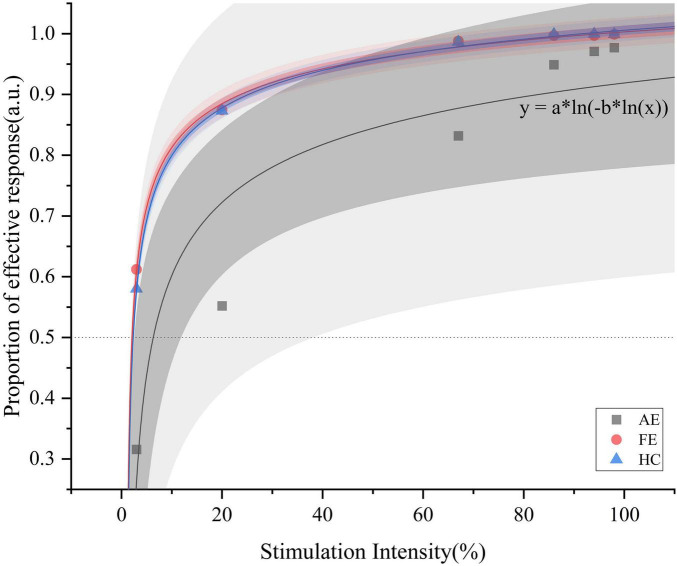
Psychometric functions with logistic fitting for the AE, FE, and HC, respectively. This figure illustrates the proportion of effective responses as a function of stimulation intensity (%) for one representative curve from each group. Data points for AE (black squares), FE (red circles), and HC (blue triangles) are shown. The darker shading indicates the 95% confidence interval, while the lighter shading represents the 95% prediction interval. The function *y* = *a* * *ln*[(−*b* * *ln*(*x*)] extends Fechner’s law [*S* = *K* * *ln*(*I*)] by introducing a nested logarithmic relationship, better capturing nonlinear response at low stimulation intensities and highlighting perceptual differences among the groups. AE, amblyopic eye; FE, fellow eye; HC, healthy control.

**FIGURE 3:**
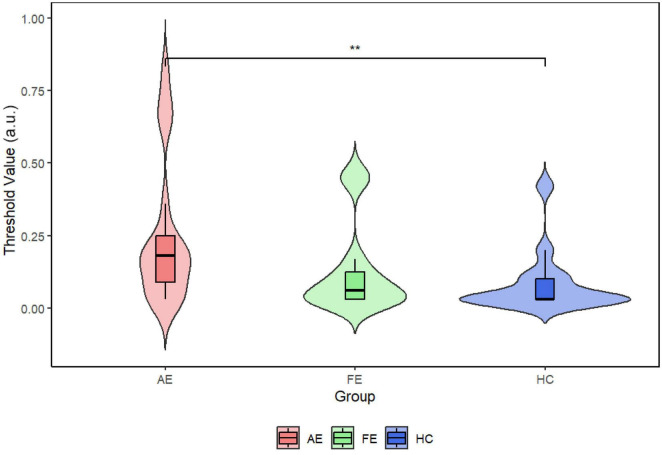
Violin plots with embedded boxplots of threshold points in each group. The Wilcoxon test showed a significant statistical difference between AE and HC (*P* = 0.0066). The mean threshold for AE was 0.24, for FE was 0.13, and for HC was 0.08. AE, amblyopic eye; FE, fellow eye; HC, healthy control. **P < 0.01.

We initially evaluated the quality of fit for all subjects suitable for fitting, observing *R*^2^ values across adult patients with amblyopia. The AE group showed an *R*^2^ of 0.885 and the FE and HC groups showed *R*^2^ values of 0.99799 and 0.99907, respectively. Figure 2 illustrates 3 representative psychophysical curves in each group. The average (± SD) thresholds for face detection were as follows (Figure 3): AE = 0.24 ± 0.25, FE = 0.13 ± 0.19, and HC = 0.08 ± 0.13, and the results showed that the face-detection threshold of the AE was significantly higher than that of the HC group (Z = 2.65, *P* = 0.008).

#### 3.4.2 Group differences in accuracy and reaction time of face-detection task across six stimulation intensity

**FIGURE 4:**
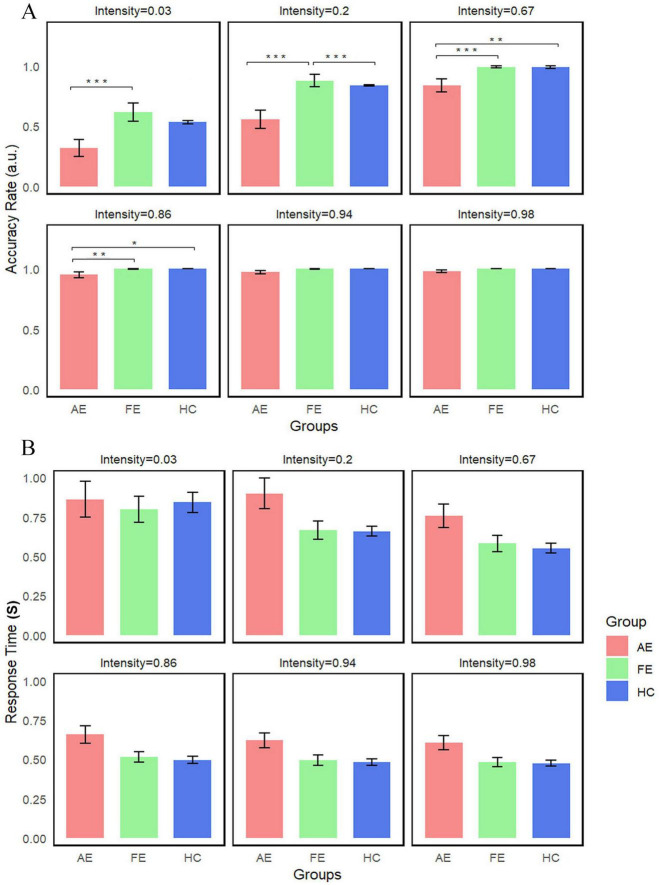
Average accuracy and reaction time of three groups in face-detection task. **(A)** Accuracy differences between groups are illustrated, with error bars representing the standard error. We indicated significant differences as follows: significant (*Ps* < 0.01) for 67 and 3% stimulus intensities (AE vs. FE); significant (*Ps* < 0.001) for 20% stimulus intensity (AE vs. FE and FE vs. HC). **(B)** No statistical difference was detected in Reaction Time. *P < 0.05; **P < 0.01, ***P < 0.001. AE, amblyopic eye; FE, fellow eye; HC, healthy control.

We conducted repeated measures Kruskal-Wallis tests with all conditions (6 levels) in each group as a within-subject factor to compare accuracy rates between the two groups. We found significant interaction effects of group × stimulus intensities for accuracy (χ^2^(2) = 27.7, *P* < 0.001) (Figure 4A). *Post-hoc* analyses revealed significant differences between AE and FE at stimulus intensities of 86% (*W* = 0, *Z* = −2.98, *P* = 0.02), 67% (*W* = 0, *Z* = −3.77, *P* < 0.001), 20% (*W* = 1, *Z* = −4.25, *P* < 0.001), and 3% (*W* = 11, *Z* = −4.01, *P* < 0.001); significant differences between AE and HC at 67% intensity (*U* = 145.5, *Z* = −3.72, *P* < 0.001); and significant differences between FE and HC at 20% intensity (*U* = 544, *Z* = 4.69, *P* < 0.001). These findings suggest substantial performance differences at these stimulus intensities. In contrast, reaction time did not show significant differences across the groups (Figure 4B).

## 4 Experiment 2: toast task

### 4.1 Stimuli

The Toast task (Figure 1D) involved 3 types of stimulation images including 2 levels stimulation intensities of 98 and 86%, as in the Face-detection task, along with pure noise images that did not contain any facial features.

### 4.2 Procedure

This experiment used a block-designed experimental paradigm that included a training period and a testing period. To fully serve its role in the training period, it was ensured that the training images would not have recognition differences, while also maintaining a distinct level of difficulty between the two intensities. As revealed by the results of the Face-detection task, we chose two intensities: 98 and 86%, each paired with pure noise images, while the testing period only involved pure noise level (240 pure noise images). Each session of the training period consisted of 120 images in total, with the ratio of face to noise images consistent with the experimenter’s semantic cues verbally. In the testing period, the experimenter provided 3 different instructions: INST1 indicated 1:1 ratio of face to noise images, INST2 indicated 3:1 ratio, and INST3 did not provide any specific ratio information as a control condition. INST1 had an image ratio set to ensure an equal representation of face and non-face images (1:1). Each eye underwent the experiment separately, experiencing two training sessions (30 difficult face images, 30 easy face images, and 60 pure noise images) and one test session (240 pure noise images). INST2 featured an adjusted image ratio to ensure a face-to-non-face image ratio of 3:1 (45 difficult face images, 45 easy face images, and 30 pure noise images) and one test experiment (240 pure noise images). To eliminate potential bias from instructions which influence decision, no semantic instruction regarding the ratio was provided in INST3, and INST3 served as a control condition, in which the image ratio (face vs. non-face) was identical to INST1.

### 4.3 Analysis

#### 4.3.1 Data analysis

Using R 4.4.1 and IBM SPSS Statistics 22, we calculated the mean and standard deviation of false alarm rate (FAR) and reaction time (RT) across the test experiment for the AE, FE in amblyopic group and HC groups at different stimulation intensities. Following the normality test, the data exhibited a non-normal distribution, this part of the analysis mirrored that of the Face-detection task.

### 4.4 Results

#### 4.4.1 FAR and RT in different groups across instructions

**FIGURE 5:**
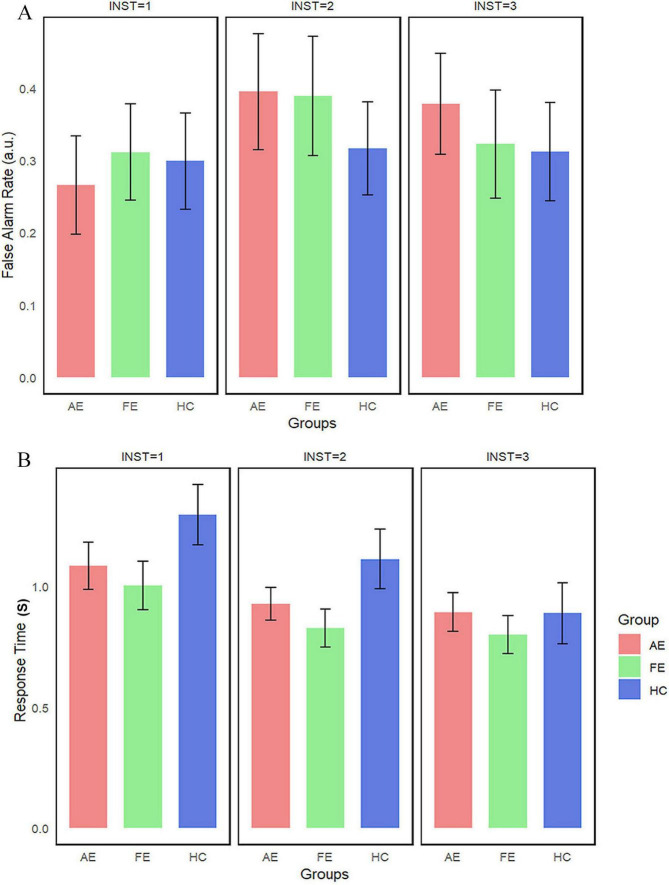
False alarm rate and reaction time in the toast task. This figures illustrate **(A)** False Alarm Rate and **(B)** Reaction Time in different groups, with error bars representing the standard error. AE, amblyopic eye; FE, fellow eye; HC, healthy control.

In the Toast task, the differences in FAR and RT are shown in Figures 5A,B. No significant statistical differences were found between the AE, FE, and HC (*Ps* > 0.1). Normality tests showed that the distribution of FAR was non-normally distributed, while RT followed a normal distribution. Consequently, we employed the Kruskal-Wallis test for the FAR and ANOVA for the RT. The results indicated that there were no statistically significant differences in FAR and RT between the groups. Descriptive statistics for FAR and RT across different instruction conditions and eye groups in the Toast task are summarized in Table 3.

**TABLE 3:** Descriptive statistics of false alarm rate and response time in the toast task.

INST	AE		FE		NE	
	**FAR (a.u.)**	**RT (ms)**	**FAR (a.u.)**	**RT (ms)**	**FAR (a.u.)**	**RT (ms)**
1	0.28 ± 0.07	1,100 ± 100	0.33 ± 0.07	1,040 ± 100	0.3 ± 0.07	1,290 ± 130
2	0.41 ± 0.08	910 ± 70	0.40 ± 0.09	830 ± 80	0.32 ± 0.07	1,110 ± 130
3	0.38 ± 0.07	890 ± 80	0.32 ± 0.07	800 ± 80	0.31 ± 0.07	890 ± 130

All data are presented as mean ± standard deviation. INST, Instruction; FAR, False Alarm Rate; RT, Reaction Time; AE, Amblyopic Eye; FE, Fellow Eye; NE, Normal Eye.

## 5 General discussion

In this study, we used Face-detection task to investigate face perception abilities in patients with amblyopia compared to HC group. Our results revealed that (1) the threshold value in the face-detection task for AE group was significantly higher than that of HC group; (2) significant differences were observed between AE group and FE group at stimulation intensities of 67, 20, and 3%, and between AE group and HC group at 67% intensity, as well as between FE group and HC group at 20% intensity.

Previous studies using similar tasks like this study have found that increased noise impairs face perception in amblyopia, which was consistent with our findings. Bankó et al. (2013b) used a similar face perception paradigm involving noise to investigate the neural mechanisms in face perception deficits in amblyopia, In this study, patients performed a 2AFC gender classification task, and the stimuli was presented either with 100% face or embedded with 50% noise. Subsequently, we introduced 2 innovations built upon this study. Firstly, we designed a Face-detection task incorporating 6 levels of intensities, guided by Fecher’s law, which allows a more detailed assessment of perceptual thresholds. Secondly, we generated pure noise stimuli using 3 different Gaussian noise, rather than applying a fixed level of noise. Banko reported that noise-induced modulation of the N170 component reflected reduced face-selective neural responses in amblyopia, which is consistent with our findings.

In the following sections, we outlined the potential mechanisms underlying impaired face perception in patients with amblyopia.

### 5.1 Noise susceptibility in amblyopia

Patients with amblyopia show a greater susceptibility to noise interference in face perception. Horovitz et al. (2004) found that N170 signals were stronger for faces than cars and decreased with added noise, especially in the right hemisphere. Significant correlations (*P* < 0.001) were observed between fMRI signals and N170 amplitudes for faces in the fusiform and superior temporal gyrus. Navajas et al. (2013) further demonstrated that N170 amplitude was linked to conscious face perception and could predict perceptual responses, while noise primarily affected the timing consistency of N170, not its amplitude. Above studies suggested that conscious face perception is associated with enhanced activity in face-selective neural regions, while noise reduces the temporal precision of this activation. We speculated that these deficits may be caused by impaired contrast sensitivity in patients with amblyopia and the crowding effect induced by noisy images.

### 5.2 Impact of contrast sensitivity on face perception

Previous studies have shown that patients with amblyopia experience reduced contrast sensitivity. Dorr et al. (2019) demonstrated that monocular and binocular contrast sensitivity deficits are defining characteristics of amblyopia. Similarly, Dulaney et al. (2023) found that fast eye movements and their amplitude were abnormal in amblyopia and correlated with both lower-order functions like contrast sensitivity and higher-order functions like optotype acuity. These findings suggest that reduced contrast sensitivity increases the perceptual difficulty of detecting faces under grayscale conditions.

### 5.3 Crowding effects in face perception

This design likely intensified the crowding effect observed in patients with amblyopia. In this study, the Reaction Time window was set to 3 s, with higher noise levels applied to images that were more difficult to recognize. Giaschi et al. (1993) examined the crowding effect in amblyopic eyes of 15 children and 15 adults with unilateral amblyopia, comparing visual acuity for high (96%) and low (11%) contrast letters in Snellen and isolated-letter formats. The results showed that amblyopia differentially affects acuity depending on contrast, with the crowding effect being stronger, weaker, or unchanged for high vs. low contrast letters in abnormal cases. In this study, we further investigated the disruptive effect of noise on face perception by introducing backgrounds composed of randomly distributed Gaussian noise dots. Unlike traditional paradigms that examine crowding effects using peripheral flankers, the noise in our design formed a globally complex visual background, thereby more closely simulating face recognition under naturalistic visual conditions. Therefore, we speculate that the crowding effect may also be one of the factors affecting Face-detection task performance in amblyopic patients in this study.

### 5.4 Impact of early visual deprivation on facial processing

In the human visual system, face perception is one of the most advanced visual skills (Moshirian Farahi et al., 2019; Yang et al., 2015). Recognizing individual identity relies on facial structures perception which remain stable despite changes in facial expressions or movements of eyes and mouth (Barabanschikov and Zherdev, 2019; Podder et al., 2023). The brain finishes face perception through contour detection, holistic processing, and spatial configuration analysis (Ahn et al., 2020; Pallett and MacLeod, 2011; Yang et al., 2014). Our study focuses on face detection, which primarily depends on low-frequency signals. When receiving facial stimulation, humans typically explore facial structures and identify the overall outline of the face. Through this step-by-step and rapid perception processing, humans can simultaneously recognize individual features and their relationships, forming a complete and identifiable representation of the face (Dobs et al., 2019; Zhang et al., 2024).

The development of face perception abilities relies heavily on early normal visual input. Previous research has demonstrated that visual deprivation during critical developmental periods can result in deficits in face perception. Kelly et al. (2012, 2019) reported impairments in both feature spacing and feature extraction aspects of face perception in patients with monocular deprivation. These results suggest an incomplete development of holistic face processing due to early monocular deprivation, which is insufficient for high-level face perception. Mondloch et al. (2013) further emphasized that early visual input is essential for the development of normal neural mechanisms for face detection. Although congenital cataract patients showed normal behavioral performance in face-detection tasks, their neural responses (P100 and N170) were abnormally large, reflecting early stage processing deficits that may lead to configural face perception impairments. In line with these findings, our study report face perception deficits in patients with amblyopia, specifically in the form of impaired face detection abilities. Our findings are consistent with studies on deprivation amblyopia, suggesting that amblyopia, as a form of early visual deprivation, may disrupt the normal development of face perception mechanisms.

In addition to cortical-level deficits, disruptions in early subcortical visual pathways may also contribute to impaired face perception in amblyopia. In addition to cortical-level deficits, disruptions in early subcortical visual pathways may also contribute to impaired face perception in amblyopia. For example, Popple and Levi (2008) reported that prolonged suppression of the amblyopic eye during childhood may disrupt normal attentional processing, and they hypothesized that this impairment may be linked to reduced connectivity between monocularly tuned early visual areas, subcortical structures involved in foveal attention, and frontal regions responsible for letter recognition and working memory. Functional MRI studies have shown reduced responses in the parvocellular layers of the lateral geniculate nucleus (LGN) and weakened feedforward connectivity from LGN to V1 when the amblyopic eye is stimulated. Although our study did not directly assess subcortical function, it is plausible that this early signal degradation limits the availability of reliable bottom-up facial information. However, the present findings alone cannot determine whether impairments in face perception are primarily driven by early signal degradation limits or by dysfunction in higher-order face-selective cortical regions such as the fusiform and occipital face areas. Future studies should include a control group in which normal subjects are presented with blurred stimuli to simulate abnormal early visual input to provide a more definitive dissociation between the effects of early sensory input and higher-order face processing, which has previously been used to minimize the influence of low-level visual features while preserving category-level distinctions in images (Gao et al., 2018).

### 5.5 Top-down modulation and compensatory attention in amblyopia

In the Toast task, no significant differences were found between amblyopic patients and normal controls in terms of false alarm rate or reaction time. One possible explanation is that semantic cues can enhance attention. This top-down expectation and selective attention may improve the accuracy of amblyopic patients, helping them compensate for their visual deficits (Ohyama and Watanabe, 2016). Supporting this, Mortazavi et al. (2022) demonstrated that both adults with amblyopia and neurotypical individuals showed higher response rates for cued targets compared to uncued ones, highlighting the role of attentional guidance. The goal of Toast task was to create prior expectations in amblyopic patients, thereby increasing their attention to target face. Results from the false alarm rate (FAR) in the Toast task suggest that amblyopic patients exhibited a higher tendency to identify stimuli as faces as the number of cues increased (e.g., INST2 and INST3). This indicates that semantic cues may have prompted amblyopic patients to rely more on attentional mechanisms, compensating for their visual deficits. However, no significant group differences were observed in our study, one possible explanation is that individuals with amblyopia may have a reduced capacity for mental imagery of faces, limiting the effectiveness of top-down guidance under ambiguous conditions. This hypothesis remains to be tested in future studies employing paradigms specifically designed to assess visual imagery capacity in amblyopia.

### 5.6 Limitations and future directions

This study incorporated 2 psychophysical tasks to assess face perception in patients with amblyopia. It is particularly innovative, offering preliminary evidence that higher-order cognitive processes may compensate for perception deficits. Our study still has several limitations. (1) The number of participants with strabismic amblyopia was not balanced with those having anisometropic and mixed amblyopia, with the former group significantly smaller than the latter two. This imbalance may limit the generalizability of the findings. (2) The results only indicate deficits in face perception ability and cannot determine whether the impairments are due to damage in one specific pathway (bottom-up or top-down) or a combined dysfunction of both. (3) Finally, although this study included participants with varying degrees of amblyopia, the sample size was insufficient to support reliable subgroup analyses. (4) Although this study investigated both bottom-up and top-down pathways using two separate tasks, it lacked a control group in which normal controls were presented with blurred stimuli to simulate abnormal early visual inputs. This limits our ability to determine whether deficits in patients with amblyopia result from early signal degeneration limits or cortical-level dysfunction. Further research with a more balanced sample and refined paradigms is needed to clarify the precise contributions of each pathway to the observed deficits in amblyopic patients and should also include a blurred vision condition in normal controls, using Gaussian-filtered face stimuli or optical blurring techniques, to directly simulate the reduced visual fidelity experienced in amblyopia.

Behavioral data alone cannot separate deficits in cognitive integration from other factors, such as attention or learning effects. Future research should use EEG to directly assess neural response which can help identify how bottom-up and top-down pathways contribute to face perception in amblyopic patients. Additionally, longitudinal studies could provide insights into whether these deficits stabilize over time or improve through targeted interventions.

## 6 Conclusion

This study provides preliminary behavioral evidence that patients with amblyopia exhibit deficits in bottom-up face perception, characterized by significantly higher detection thresholds and lower recognition accuracy compared to normal controls. The findings suggest a complex interaction between bottom-up and top-down processes, where higher-order cognitive mechanisms may enhance perceptual performance in challenging contexts. Future studies should incorporate larger sample sizes, neuroimaging techniques and blurred-stimulus control conditions to clarify whether deficits in face perception primarily from early signal input limits or cortical dysfunction and further elucidate the neural basis of these compensatory mechanisms and their potential for targeted therapeutic interventions.

## Data Availability

The original contributions presented in this study are included in this article/Supplementary material, further inquiries can be directed to the corresponding authors.
