# Supplementary material for: Psychophysical assessment of face perception deficits in adults with amblyopia through top-down and bottom-up visual processing pathways
Source: Front Neurosci. 2025 May 21;19:1548243. doi: 10.3389/fnins.2025.1548243 (PMC12133779; doi:10.3389/fnins.2025.1548243)
Supplement: Supplementary file 1 [file Supplementary_file_1.docx]

**Supplementary materials**

*S1 sample size calculation in two tasks*

**Face-detection task**

The sample size for the face-detection task was determined using the power analysis approach. We aimed to detect a significant difference in the primary outcome measures between patients with amblyopia and healthy controls (HC) with a two-tailed significance level of 0.05. Based on previous literature, we estimated the effect size (Cohen's d) to be approximately 0.5, indicating a medium to large effect. Using G*Power software, we calculated the required sample size to achieve a statistical power of 0.80. The analysis indicated that a total of 50 participants (25 in each group) would be necessary to detect the expected effect size with the desired power. This sample size was confirmed to be adequate after accounting for possible participant dropout and deviations from the study protocol, ensuring that enough participants would complete the study. In our preliminary experiment, the overall data exhibited a non-normal distribution. Consequently, we chose the Wilcoxon signed-rank test as the appropriate statistical method for analysis.

Thus, the final cohort (**See Figure S1**) for the Face-detection task comprised 25 patients with amblyopia and 25 healthy controls, aligning with our pre-defined sample size calculation parameters.


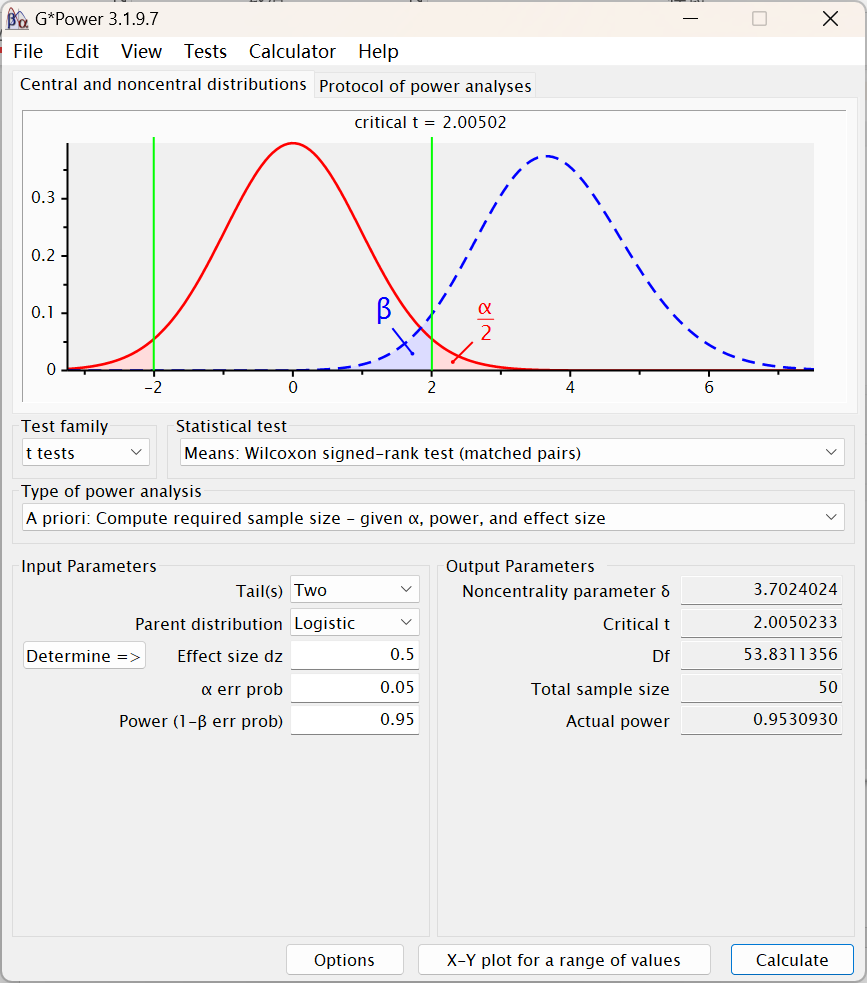


**Figure S1**. Sample size calculation process for the Face-detection task. This figure illustrates the power analysis performed using G*Power software, indicating the necessary sample size to achieve a statistical power of 0.95 for detecting significant differences between patients with amblyopia and healthy controls.

**Toast task**

The sample size for the toast task was impacted by participant dropout following the completion of the initial face-detection task. As a result (**See Figure S2**), only 16 patients with amblyopia and 15 healthy controls were included in the analysis. Despite the smaller size, post-hoc power analysis indicated that this sample size of 31 participants (16 patients and 15 HCs) was sufficient to maintain a statistical power of 0.80 for detecting significant differences between the groups.

**
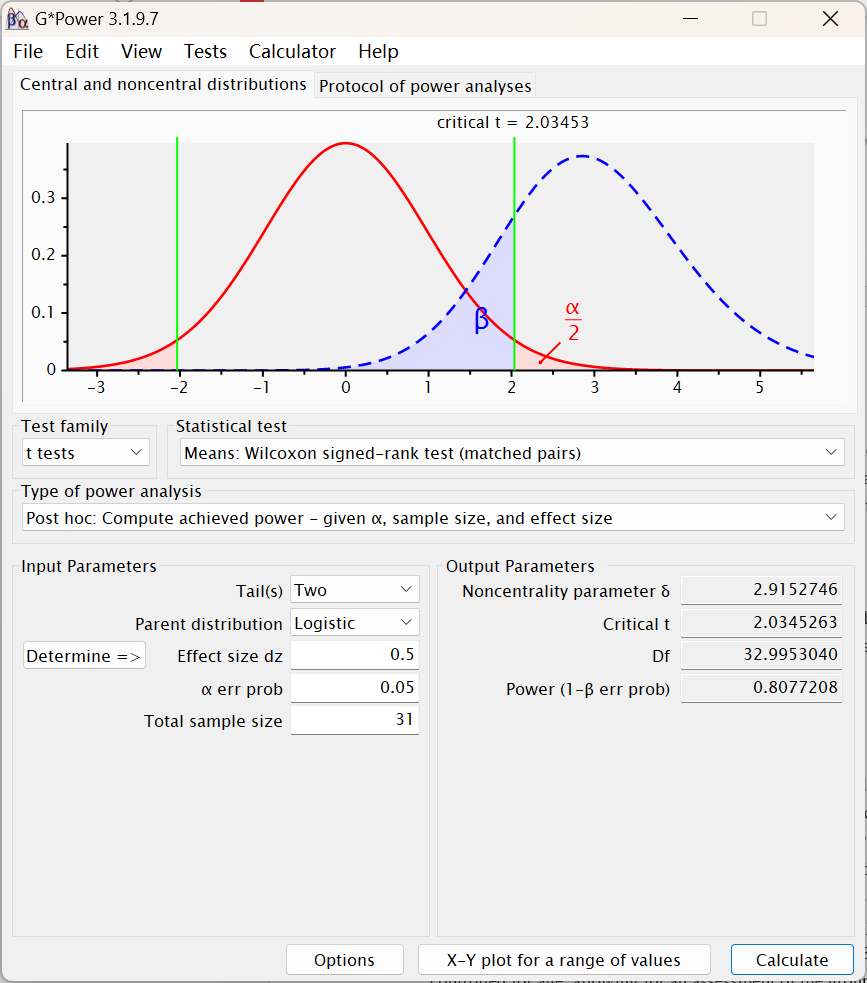
**

**Figure S2**: Statistical power analysis for the Toast task. This figure illustrates that with a sample size of 31 participants (16 patients and 15 healthy controls), the statistical power remains at 0.80.

*S2 Age-stratification calculations*

**Methods**

Since both eyes in the healthy control (HC) group showed similar performance, the averaged result from both eyes were used in all analyses. There is a significant difference in age between amblyopic patients and normal subjects in the Face-detection task (*P* = 0.017). To assess the impact of age on the accuracy rate (ACC) of each group, we conducted a stratified analysis of the data. The participants were first categorized into two age groups: 18-27 years and 28-36 years. Subsequently, a Kruskal-Walli’s test was applied within each age group and stimulus intensity combination to compare the ACC differences among the three groups (AE, FE, and HC). This approach effectively controlled for age, allowing for an assessment of the group effect on ACC. By analyzing the results of the Kruskal-Wallis test for each combination, we identified significant differences between the groups, thereby providing a foundation for further analyses.

**Table S1**. Stratified analysis of accuracy (ACC) differences between amblyopic patients and healthy controls (HCs) across various stimulus intensities and age groups.

| Age Group | Stimulus Intensity | Kruskal-Walli’s chi-squared | df | p-value |
| --- | --- | --- | --- | --- |
| 28-36 | 0.98 | 3.0280 | 2 | 0.22 |
| 28-36 | 0.94 | 2.7581 | 2 | 0.25 |
| 28-36 | 0.86 | 1.6336 | 2 | 0.44 |
| 28-36 | 0.67 | 4.1748 | 2 | 0.12 |
| 28-36 | 0.20 | 6.4572 | 2 | 0.039 |
| 28-36 | 0.03 | 4.8247 | 2 | 0.089 |
| 19-27 | 0.98 | 2.4078 | 2 | 0.30 |
| 19-27 | 0.94 | 3.4472 | 2 | 0.18 |
| 19-27 | 0.86 | 13.0790 | 2 | 0.0014 |
| 19-27 | 0.67 | 15.7020 | 2 | 0.00039 |
| 19-27 | 0.20 | 15.2400 | 2 | 0.00049 |
| 19-27 | 0.03 | 6.9953 | 2 | 0.030 |
| <18 | 0.98 | 1.0000 | 1 | 0.32 |
| <18 | 0.94 | NaN | 1 | NaN |
| <18 | 0.86 | 1.0000 | 1 | 0.32 |
| <18 | 0.67 | 1.0000 | 1 | 0.32 |
| <18 | 0.20 | 1.0000 | 1 | 0.32 |
| <18 | 0.03 | 1.0000 | 1 | 0.32 |

**Results**

For the stratified analysis (**Table S1**), significant differences in accuracy (ACC) between amblyopic patients aged 19-27 and HCs were observed at stimulus intensities of 0.86 (*P* = 0.0014), 0.67 (*P* = 0.00039), 0.03 (*P* = 0.030), and 0.2 (*P* = 0.00050). In addition, a significant difference was found at a stimulus intensity of 0.2 between patients aged 28-36 and HCs (*P* = 0.040). These findings indicate that the differences of ACC among the groups remain statistically significant with age as a covariate.
